# Deep neck infections: A single-center analysis of 63 cases

**DOI:** 10.4317/medoral.21799

**Published:** 2017-08-16

**Authors:** Philipp Kauffmann, Robert Cordesmeyer, Markus Tröltzsch, Christian Sömmer, Rainer Laskawi

**Affiliations:** 1 MD DMD, Department of Oral and Maxillofacial Surgery, University of Göttingen, Germany; 2 MD DMD, Department of Otolaryngology-Head and Neck Surgery, University of Göttingen, Germany; 3 MD PhD Department of Otolaryngology-Head and Neck Surgery, University of Göttingen, Germany

## Abstract

**Background and Purpose:**

With the use of antibiotic therapy, the incidence of deep neck infections has decreased in recent decades. The aim of this investigation was to review the clinical course and the management of deep neck infections in our department, compare them to the experiences of the common literature and identify predisposing factors for lethal complications.

**Material and Methods:**

In this single-center analysis, 63 patients with deep neck infections were treated surgically. The following clinical data were analyzed and compared: age, gender, laboratory data, spatial manifestation, therapeutic modalities, comorbidities, length of hospitalization and complications.

**Results:**

There was a predominance of male patients (58.7%) and a mean age of 57.9 years. The most common symptoms at diagnosis were sore throat (96.8%) and neck swelling (92.0%). Cardio/pulmonary diseases and diabetes mellitus were the most common comorbidities. There was a significantly longer hospital stay for patients with diabetes mellitus. The most common manifestation was a parapharyngeal abscess in 24 patients (38.1%), followed by peri-/retrotonsillar infections in 19 patients (30.2%). In 29 patients, a multiple space infection was observed, with a significantly longer duration of hospitalization and a higher rate of complications. The main life-threatening complication was the development of airway obstruction in 20 patients (31.7%), who all received a tracheostomy. The duration of hospitalization for patients with complications was significantly longer.

**Conclusions:**

Close attention must be paid to the management of patients with deep neck infections, especially patients with diabetes mellitus and cardio/pulmonary diseases or patients with multiple space infections.

** Key words:**Deep neck infections, comorbidities, surgical treatment, tracheostomy, diabetes mellitus.

## Introduction

Deep neck infections are defined as infections in the potential fascial planes and spaces of the head and neck. The anatomy of the head and neck is complex, with many spaces that are connected to each other. The incidence of deep neck infections is decreasing with the use of antibiotics. However, they may still lead to lethal complications, such as airway obstruction, mediastinitis or septic shock (1). The management of deep neck infections is challenging and should be carried out by an interdisciplinary team. Dental infections are described as the most common cause of deep neck infections in adults and acute tonsillitis in children (2). The prognosis of deep neck infections depends on the comorbidities of the patient, such as diabetes mellitus, alcoholism or drug abuse (3). Computer tomography (CT-scan) of the head and neck and the upper thorax is the most common tool for diagnosis (4). However, the occurrence of deep neck infections is a serious situation that can be associated with life threatening complications and high mortality. Therapy is challenging and can only be achieved through an interdisciplinary treatment concept based on experience and knowledge.

## Material and Methods

The study was approved by the ethics committee of the University of Göttingen (6/7/13 An). Data collection included surgically treated patients with deep neck infections, including retro- and parapharyngeal abscess, head and neck phlegmona and necrotizing fasciitis.

The observation period lasted from 01.01.2002 until 31.12.2012 and included 63 patients, who were all treated in the university medical center of Göttingen. They all received a CT-scan. Only cases with surgically treated deep neck infections were included in the study. Patients with infected head and neck tumors, superficial skin abscesses, iatrogenic or posttraumatic neck infections and patients with incomplete data were excluded from the analysis.

Personal medical data, such as age and sex, abscess location, laboratory results, treatment, diagnosis, complications and hospitalization time, were assessed.

The reference ranges for standard values for laboratory data were 4.0-11.0 thousands per µL for total leucocyte count, <5.0 (mg/l) for C-reactive protein (CRP) and 13.5-17.5 (g/dl) for hemoglobin (Hb). Depending on sex, a value under 13.5 g/dl (male) or 12.0 g/dl (female) was defined as anemia.

Statistical analysis was performed with the program STATISTICA for Windows, version 10.0 (StatSoft, Inc., Germany). For pairwise comparisons, the Mann-Whitney U-test was used, and for the categorical variables, the exact Fisher test was used. The significance level was *p* = 0.05.

## Results

- Demographic and clinical data

**Figure 1:**
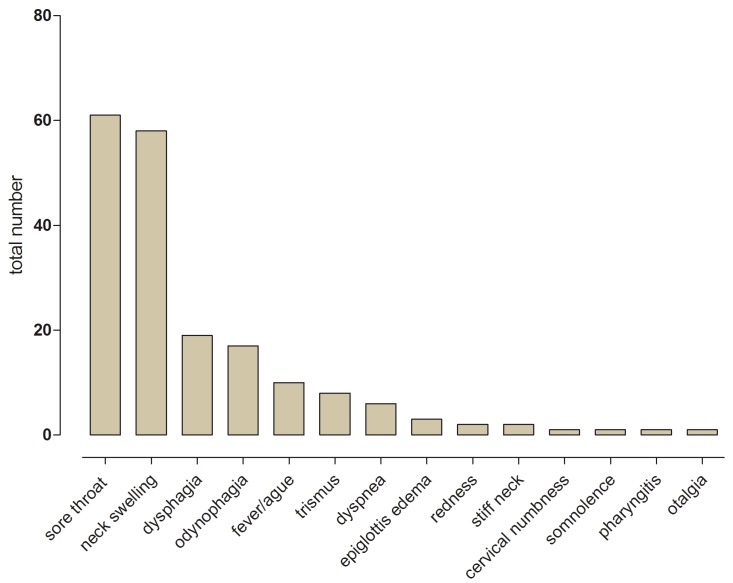
Presenting total number of different symptoms at time of diagnosis.

A total of 37 of the 63 patients were male (58.7%) and 26 were female (41.3%), ranging in age from 19 to 97 years (median 57 years). The most common symptoms at diagnosis were sore throat (96.8%) and neck swelling (92.0%). Other symptoms were dysphagia (30.2%), odynophagia (26.9%), fever (15.9%), trismus (12.7%) and airway obstruction (9.5%). Further symptoms can be found in Figure 1.

The total leucocyte count (reference number 4.0-11.0 thousands per µL ) was increased in 45 cases (71.4%), with a mean of 15.4 thousands per µL (range from 4.0 thousands per µL to 31.5 thousands per µL). The mean CRP was 156.2 mg/l (range from 2 mg/l to 472.7 mg/l), and the mean hemoglobin was 13.1 g/dl (range from 7.2 g/dl to 16.9 g/dl). A total of 29 patients (46.0%) showed anemia, according to the different reference values for male and female patients. In our analysis, the laboratory parameters in diabetic and non-diabetic patients were significantly different only at the hemoglobin level, which in the diabetic patients was in the range for anemia (10.8 g/dl vs. 13.5 g/dl). A significant impact of diabetes mellitus on the CRP and leukocytes could not be observed, but a tendency was found.

- Comorbidities

**Figure 2:**
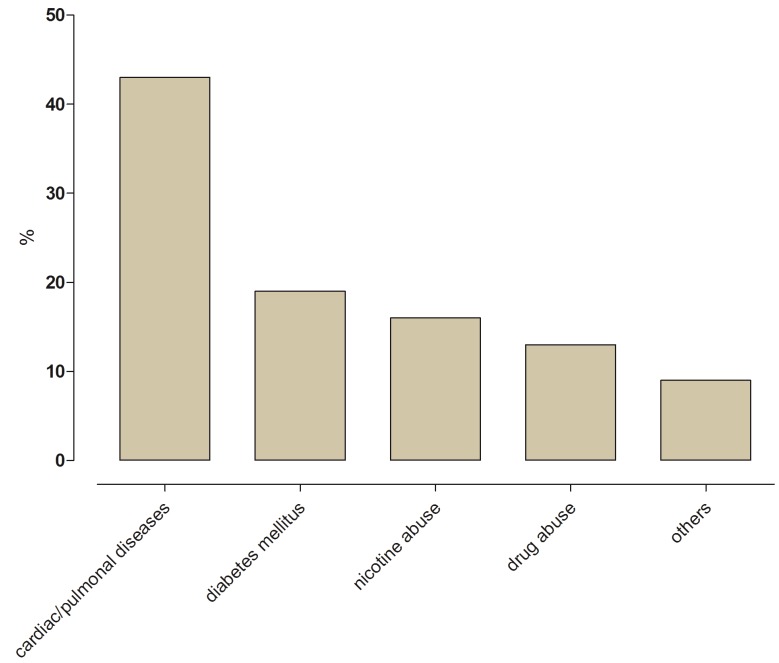
Presenting number of comorbidities in percent (%).

One comorbidity was found in 23 (56.1%) cases, and two or more comorbidities were found in 18 cases (43.9%). The most prevalent comorbidities were cardio/pulmonary diseases (43.0%), diabetes mellitus (19.0%), nicotine consumption (16.0%), intravenous drug injection (13.0%) and others (9.0%) (Figure 2). Diabetic patients in our study had a significantly longer duration of hospital stay compared to non-diabetic patients (21.9 days vs. 13.7 days) ( Table 1).

**Table 1:**
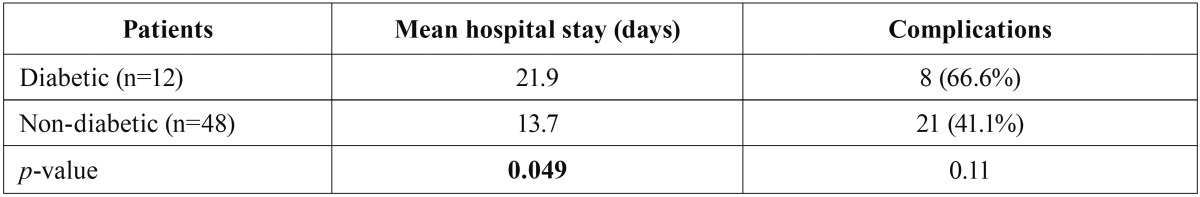
Comparison between diabetics and non-diabetics regarding hospital stay and complications, a significance level of *p*≤ 0,05 was selected.

 - Etiology and spaces

The most common manifestation was a parapharyngeal abscess in 24 cases (38.1%), followed by peri-/retrotonsillar infections in 19 cases (30.2%) and retropharyngeal abscesses in 7 (11.1%) ( Table 2). The infection occurred in 51.0% on the left side, 46.0% on the right side and 3.0% on both sides. In 29 cases (46.1%), multiple space involvement was observed. The average duration of hospitalization time and the rate of complications were significantly longer and higher in patients with multiple space involvement ( Table 3). As an inflammatory parameter, the CRP value was significantly higher in patients with multiple abscesses in various spaces (202.7 mg/l vs. 116.5 mg/l) ( Table 4).

**Table 2:**
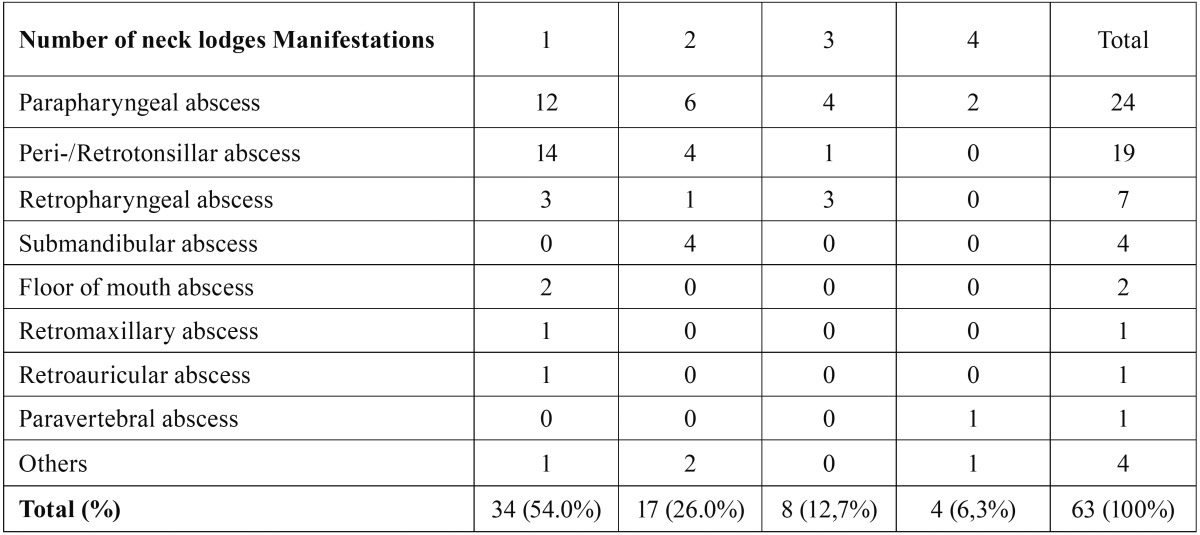
The table shows a list with the number of the affected spaces.

**Table 3:**
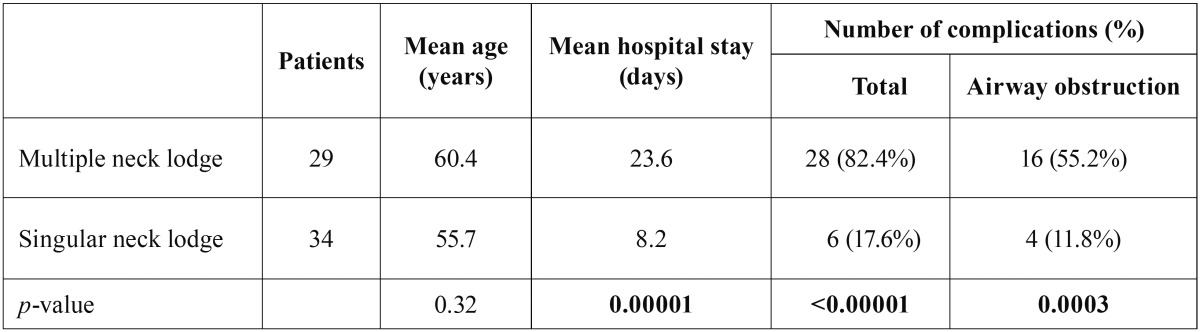
Statistical comparison between multiple neck lodges vs. singular neck lodges regarding mean age, mean hospital stay (days) and number of complications in percent (%) including airway obstruction, a significance level of *p*≤ 0,05 was selected.

**Table 4:**
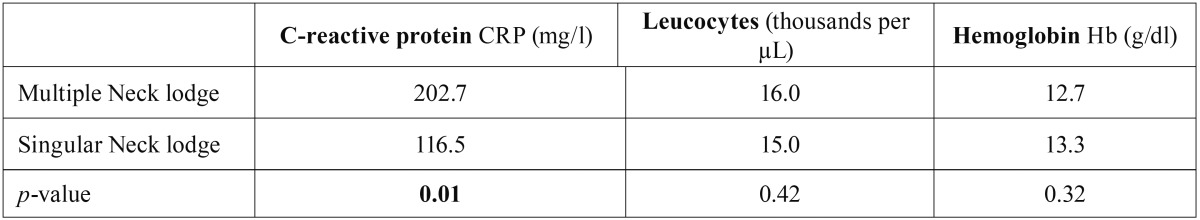
Results of the statistical comparison between multiple neck lodges vs. singular neck lodges regarding the laboratory chemical data C-reactive protein (CRP mg/l), leucocytes (thousands per µL ) and hemoglobin (Hb g/dl), a significance level of *p*≤ 0,05 was selected.

- Treatment

All patients were treated empirically with broad-spectrum intravenous antibiotics on admission. The first-line therapy was modified according to microbiological findings. The most frequent treatment regimes, alone or in combination, were amoxicillin/clavulanate potassium (62.0%), second- and third-generation cephalosporins (14.3%) and clindamycin (4.7%). In 33 patients, the first-line antibiotic therapy was successful; in 21 cases, the antibiotic spectrum had to be escalated. In 9 cases, there was total resistance to the first-line antibiotic, and there was a significantly longer duration of hospitalization (13 days, *p*=0.019) for these patients.

All 63 patients underwent an open surgical drainage under general anesthesia. An external transcervical approach was necessary in 51 cases (81.0%), transoral in 4 cases (6.4%) and a combined approach in 8 cases (12.7%). A wide exposure of the abscess cavity and debridement of necrotic tissue was performed. In 7 cases, there was a second revision operation necessary (11.1%). Of these 7 patients, 6 had a multiple space process.

- Complications and mortality

The main life-threatening complication was the development of an airway obstruction, which was seen in 20 cases (31.7%). All twenty patients received a tracheostomy. Mediastinitis was seen in 17.5% and necrotizing fasciitis in 12.5%. Further complications, such as septic shock, pneumonia or lung emboli were seen in 4.8%. There were 2 deaths by septic shock and lung emboli (mortality rate 3.0%).

The average hospital stay of patients without comorbidities was 10.0 days. The duration of hospitalization of patients with complications was significantly longer. Patients with airway obstruction stayed 26.7 days, with mediastinitis, 20.6 days and with necrotizing fasciitis, 21.7 days.

In regards to the development of complications, 66.6% of diabetic patients developed a complication, and only 41.1% of non-diabetic patients did; although there was a trend, the difference was not significant.

## Discussion

The patient population of this work is in its composition comparable to that of other studies (5). With 37 of 63 patients (58.7%) being male, a modest predominance of men was observed. The age ranged from 19 to 97 years, and the median age of our patient population was 58 years, which is comparable to another analysis (6).

Deep neck infections were seen in 24 patients (38.1%) and were most commonly located in the parapharyngeal space. This result is consistent with other studies (7) and is anatomically reasonable, as the parapharyngeal space communicates with other spaces (8).

In our analysis, 29 (46.1%) of 63 patients showed a multiple space infection. It was found that multiple space infections are accompanied by a significantly higher incidence of complications (82.4% vs. 6.0%). Airway obstruction occurred significantly more frequently in multiple space infections (11.8% vs. 55.2%). The results of other studies are similar (6).

The frequency of symptoms in our patient cohort is consistent with the present literature of deep neck infections. Several authors found a sore throat, as well as a throat swelling and dysphagia with pain and fever (9,10). However, if respiratory distress exists, this is a sign of a serious complication of deep neck infection (11).

The CRP value during hospitalization averaged 156.2 mg/l and is similar to those in the literature (4,6,11). Of the patients, 71.4% had leukocytosis at admission, which is comparable to findings in other studies (12). Srivanitvhapoom *et al.* (13) reported in their study that most of the patients had normal Hb values and pathological leukocyte values at the same time. All three laboratory parameters mentioned are indeed non-specific, but together are well suited for follow-up in these cases.

In our patient population, comorbidities could be detected in 65.1% of cases (41 patients), including 18 patients (43.9%) with two or more disorders. Umeda *et al.* (14) reported in their literature review that 56.0% of the patients had comorbidities, such as diabetes mellitus (24.8%) or alcohol abuse (16.8%). Srivanitchapoom *et al.* (13) also examined the influence of comorbidities on the hemoglobin level and could find no influence, in contrast to our results. Zheng *et al.* (15) examined clinical features of deep neck infections comparing diabetic and non-diabetic patients and found, just as we did, no significant effect on the leukocyte value. However, they could also show a trend, as the number of neutrophils was significantly higher in diabetic patients.

Diabetic patients had a significantly longer duration of hospitalization compared to non-diabetic patients (21.9 days vs. 13.7 days). Such results can also be found in the international literature (16,17). Hunag *et al.* and Rao *et al.* (18,19) have implicated poor immune status as the ultimate cause in the development of complications and the further course in deep neck infections.

The mainstay of treatment for deep neck infections is adequate surgical drainage of the abscess cavity coupled with appropriate antibiotic coverage and securing the airway (20-23).

All of our patients received a surgical intervention. In literature, the operation rate in deep neck infections is lower at values between 60 to 100% (11,24). Here, the various surgical approaches should depend on the location and extent of the abscess (25). A study by Boscolo Rizzo *et al.* (6) showed that nearly two-thirds of patients suffering from a deep neck infection had a successful response to treatment solely comprising antibiotics. In selected patients, they administered intravenous antibiotic treatment, coupled with an aggressive contrast-enhanced CT-based “wait-and-watch” approach and were able to avoid surgical drainage.

Tracheotomy is often necessary in an obstruction of the upper airways. In our study, 31.7% of the cases (20 patients) needed a tracheotomy as a lifesaving emergency measure. Compared to the studies of Agarwal *et al.* (9) and Larawin *et al.* (26), with 12% and 8.7%, our tracheotomy incidence is higher. In our patient population, 11.1% of the cases had to be re-operated. Of these, 85.7% had a multiple space manifestation. Our reoperation rate from 11.1% is comparable with other series, which reported rates of 3 to 14.1% (27,28).

The most common complications were airway obstruction (31.7%), mediastinitis (17.5%) and necrotizing fasciitis (12.7%). Other studies also report that an obstruction of the upper respiratory tract is the most common complication (3,11). According to the international literature, the mortality rate following mediastinitis is from 9 to 50%, and that of necrotizing fasciitis is 40-76% (29). Overall, two deaths occurred (3.1%). According to Santos Gorjón *et al.* (30), the mortality rate from deep neck infections is approximately 4.9% in adults and 6.2% in children.

The average duration of hospitalization was 15.3 days in our patient population. This is comparable to durations reported by other studies (9,27).

According to Lee and Kanagalingam (7), it is statistically proven that patients experiencing a complication and elderly patients have a significantly longer duration of hospitalization. The same result was confirmed by our analysis. Patients with advanced complications, especially airway obstruction, descending mediastinitis or necrotizing fasciitis, had a significantly greater length of hospital stay.

Close attention must be paid to the management of patients with deep neck infections, especially patients with diabetes mellitus and cardio/pulmonary diseases or patients with multiple space infections.
